# Crystal structure of (*S*)-4-carbamoyl-4-(1,3-dioxo­isoindolin-2-yl)butanoic acid^1^

**DOI:** 10.1107/S2056989014027121

**Published:** 2015-01-01

**Authors:** Kohei Otogawa, Kazuhiko Ishikawa, Motoo Shiro, Toru Asahi

**Affiliations:** a Graduate school of Advanced Science and Engineering, Waseda University (TWIns), Tokyo 162-8480, Japan; b Consolidated Research Institute for Advanced Science and Medical Care, Waseda University (ASMeW), Tokyo 162-0041, Japan

**Keywords:** crystal structure, thalidomide, hydrogen bonds

## Abstract

In the title compound, C_13_H_12_N_2_O_5_, the phthalimide ring system is essentially planar, with a maximum deviation of 0.0479 (14) Å. In the crystal, each mol­ecule is linked *via* six neighbouring mol­ecules into a three-dimensional network through N—H⋯O and O—H⋯O hydrogen bonds, which form an *R*_3_^2^(8) ring motif.

## Abstract 2

The title compound is a one of the first-step hydrolysis products of thalidomide. In the crystal, each mol­ecule is linked *via* six neighbouring mol­ecules into a three-dimensional network through N—H⋯O and O—H⋯O hydrogen bonds.

## Chemical context   

The title compound, 5-(amin­oxy)-4-(3-oxo-2*H*-isoindol-2-oyl)valeric acid (phthaloylisoglutamine), is one of the first-step hydrolysis products of thalidomide. Thalidomide was first synthesized in 1953 and was marketed as a hypnotic medicine in 1956. After that, the teratogenic side effect of thalidomide was proved and caused serious drug disaster (Lenz, 1961 ▸). Blashke *et al.* (1979 ▸) reported that only (*S*)-thalidomide exhibits teratogenicity while (*R*)-thalidomide exhibits sedative effects. In other words, the hypnotic and teratogenic mechanisms of thalidomide are different. For a long time, the target protein of thalidomide has not been clarified. However in 2010, the protein cereblon, which is one of the E3 ubiquitin ligase proteins, was identified as the primary target of thalidomide teratogenicity (Ito *et al.*, 2010 ▸). Furthermore, the conformation of a Cereblon and thalidomide complex has been reported (Fischer *et al.*, 2014 ▸).
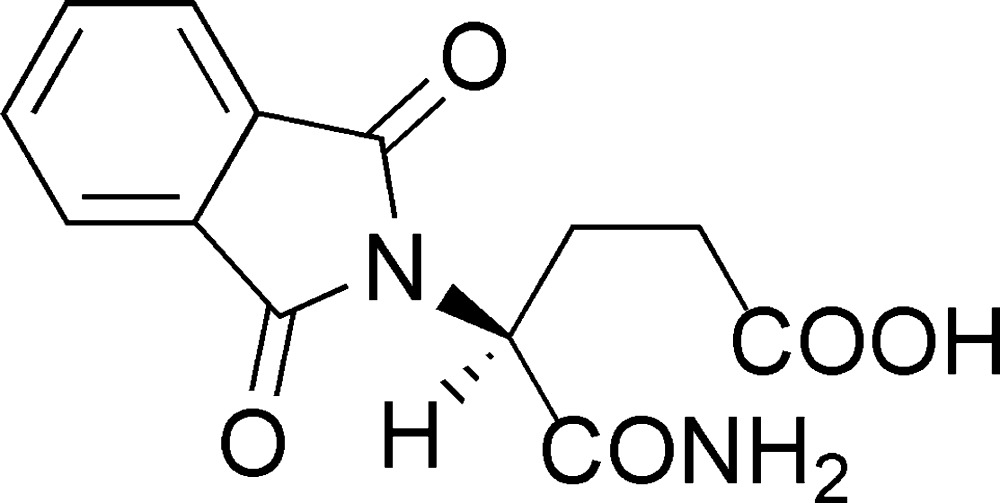


Hydrolysis compounds of thalidomide are generated rapidly *in vivo* (Schumacher *et al.*, 1965 ▸; Nishimura *et al.*, 1994 ▸) and some of these showed TNF-α production-inhibitory activity (Nakamura *et al.*, 2007 ▸). Although the crystal structures of racemic and enanti­omeric thalidomide were solved and reported earlier (Allen & Trotter, 1971 ▸; Suzuki *et al.*, 2010 ▸), the crystal structures of hydrolysis compounds of thalidomide have not been reported. Considering that knowing the structure of the mol­ecule enables us to calculate the affinity between ligand and receptor using computer simulation, our report herein will be helpful in clarifying the differences between the biological effects of thalidomide and phthaloylisoglutamine.

## Structural commentary   

**Figure 1:**
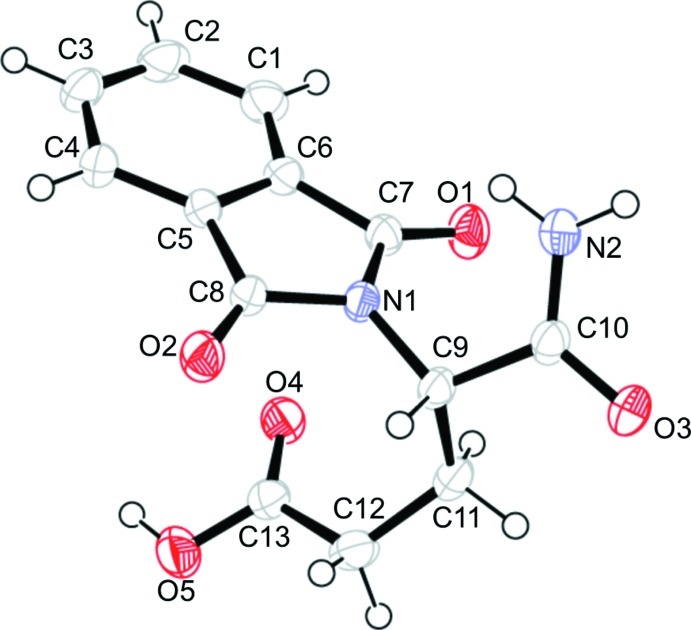
The mol­ecular structure of the title compound, showing displacement ellipsoids at the 50% probability level.

**Figure 2:**
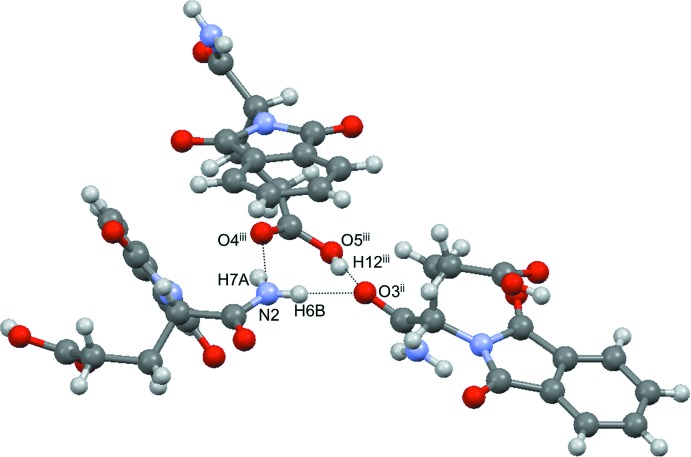
A trimer structure of the title compound and an 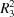
(8) ring motif formed through O5^iii^—H12^iii^⋯O3^ii^, N2—H6*B*⋯O3^ii^ and N2—H7*A*⋯O4^iii^ hydrogen bonds. [Symmetry codes: (ii) *x* + 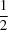, −*y* + 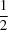, −*z* + 1; (iii) −*x* + 1, *y* − 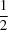, −*z* + 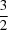.]

The mol­ecular structure of the title mol­ecule is shown in Fig. 1 ▸. The asymmetric center is *S* for atom C9. The phthalimide ring (N1/C1–C8) is essentially planar, with a maximum deviation of 0.0479 (14) Å for N1. The carbon–oxygen distances in the carb­oxy group (COOH) show different lengths [C13—O4 = 1.206 (2) and C13—O5 = 1.316 (2) Å]. This difference indicates that the C—O bonds in the carb­oxy group are non-delocalized. These bonds are slightly strengthened by inter­molecular O5—H12⋯O3 and O4⋯H7*A*—N2 hydrogen bonding (Fig. 2 ▸). The conformation of C9—C11—C12—C13 chain is slightly twisted *gauche* [torsion angle = 77.4 (2)°].

## Supra­molecular features   

**Figure 3:**
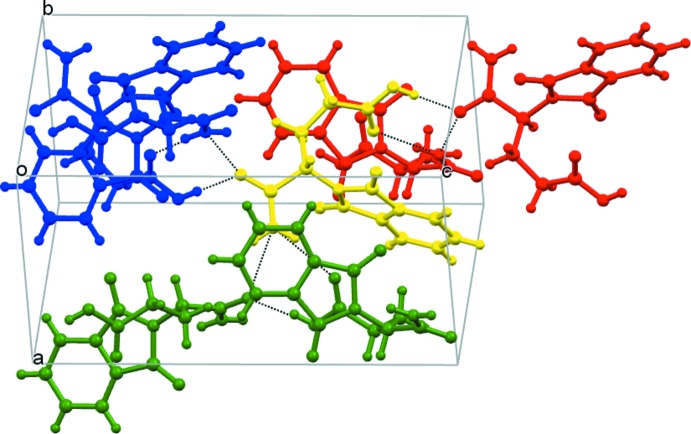
A crystal packing view of the title compound, showing the inter­molecular hydrogen bonds. A yellow mol­ecule is linked with two red, two green and two blue mol­ecules.

In the crystal structure, each mol­ecule has six hydrogen bonds, which are divided into three types (Table 1 ▸). The three hydrogen bonds form a hydrogen-bonded ring with an 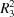
(8) ring motif, which unites three mol­ecules (Fig. 2 ▸). Taken together as shown in Fig. 3 ▸, one mol­ecule (yellow) is linked to another six mol­ecules (blue, red, and green) by three sets of circular hydrogen bonds.

## Database survey   

A search of the Cambridge Structural Database (Version 5.35 update in 2014; Groom & Allen, 2014 ▸) for the structure of thalidomide gave 11 hits, but there was no hydrolysis compound of thalidomide.

## Synthesis and crystallization   

The title compound was purchased from WuXi AppTec. The title compound (2 mg) was dissolved in ethanol (500 µl). After a few days of slow evaporation at 278 K, colourless single crystals suitable for X-ray diffraction were obtained.

## Refinement   

Crystal data, data collection and structure refinement details are summarized in Table 2 ▸. All H atoms were included in calculated positions [C—H (aromatic) = 0.93, C—H (methine) = 0.98, C—H (methyl­ene) = 0.97, N—H = 0.87 and O—H = 0.82 Å] and treated as riding atoms with *U*_iso_(H) = 1.2*U*_eq_(C) and 1.5*U*_eq_(N, O).

## Supplementary Material

Crystal structure: contains datablock(s) global, I. DOI: 10.1107/S2056989014027121/is5381sup1.cif

Structure factors: contains datablock(s) I. DOI: 10.1107/S2056989014027121/is5381Isup2.hkl

Supporting information file. DOI: 10.1107/S2056989014027121/is5381Isup3.cml

CCDC reference: 1038803

Additional supporting information:  crystallographic information; 3D view; checkCIF report

## Figures and Tables

**Table 1:** Hydrogen-bond geometry (, )

*D*H*A*	*D*H	H*A*	*D* *A*	*D*H*A*
O5H12O3^i^	0.83	1.80	2.6230(19)	172
N2H6*B*O3^ii^	0.87	2.32	2.891(2)	123
N2H7*A*O4^iii^	0.87	2.05	2.886(2)	161

Symmetry codes: (i) 
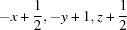
; (ii) 
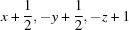
; (iii) 
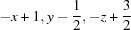
.

**Table 2:** Experimental details

Crystal data	
Chemical formula	C_13_H_12_N_2_O_5_
*M*_r_	276.25
Crystal system, space group	Orthorhombic, *P*2_1_2_1_2_1_
Temperature (K)	223
*a*, *b*, *c* ()	8.4790(3), 9.6751(3), 15.4488(5)
*V* (^3^)	1267.35(7)
*Z*	4
Radiation type	Cu *K*
(mm^1^)	0.96
Crystal size (mm)	0.63 0.20 0.10

Data collection	
Diffractometer	Rigaku R-AXIS RAPID
Absorption correction	Multi-scan (*ABSCOR*; Rigaku, 1995 ▸)
*T*_min_, *T*_max_	0.766, 0.908
No. of measured, independent and observed [*F* ^2^ > 2(*F* ^2^)] reflections	23228, 2320, 2245
*R*_int_	0.058
(sin /)_max_ (^1^)	0.602

Refinement	
*R*[*F* ^2^ > 2(*F* ^2^)], *wR*(*F* ^2^), *S*	0.027, 0.067, 1.08
No. of reflections	2320
No. of parameters	183
H-atom treatment	H-atom parameters constrained
_max_, _min_ (e ^3^)	0.16, 0.16
Absolute structure	Flack *x* determined using 914 quotients [(*I* ^+^)(*I* )]/[(*I* ^+^)+(*I* )] (Parsons *et al.*, 2013 ▸)
Absolute structure parameter	0.06(4)

Computer programs: *RAPID-AUTO* (Rigaku, 1998 ▸), *SHELXS2013* and *SHELXL2013* (Sheldrick, 2008 ▸) and *CrystalStructure* (Rigaku, 2014 ▸).

## supplementary crystallographic information

### Crystal data

**Table:**

C_13_H_12_N_2_O_5_	*D*_x_ = 1.448 Mg m^−^^3^
*M**_r_* = 276.25	Cu *K*α radiation, λ = 1.54187 Å
Orthorhombic, *P*2_1_2_1_2_1_	Cell parameters from 12101 reflections
*a* = 8.4790 (3) Å	θ = 4.6–68.3°
*b* = 9.6751 (3) Å	µ = 0.96 mm^−^^1^
*c* = 15.4488 (5) Å	*T* = 223 K
*V* = 1267.35 (7) Å^3^	Needle, colorless
*Z* = 4	0.63 × 0.20 × 0.10 mm
*F*(000) = 576.00	

### Data collection

**Table:**

Rigaku R-AXIS RAPID diffractometer	2245 reflections with *F*^2^ > 2σ(*F*^2^)
Detector resolution: 10.000 pixels mm^-1^	*R*_int_ = 0.058
ω scans	θ_max_ = 68.2°, θ_min_ = 5.4°
Absorption correction: multi-scan (*ABSCOR*; Rigaku, 1995)	*h* = −10→10
*T*_min_ = 0.766, *T*_max_ = 0.908	*k* = −11→11
23228 measured reflections	*l* = −18→18
2320 independent reflections	

### Refinement

**Table:**

Refinement on *F*^2^	H-atom parameters constrained
*R*[*F*^2^ > 2σ(*F*^2^)] = 0.027	*w* = 1/[σ^2^(*F*_o_^2^) + (0.0326*P*)^2^ + 0.1235*P*] where *P* = (*F*_o_^2^ + 2*F*_c_^2^)/3
*wR*(*F*^2^) = 0.067	(Δ/σ)_max_ < 0.001
*S* = 1.08	Δρ_max_ = 0.16 e Å^−^^3^
2320 reflections	Δρ_min_ = −0.16 e Å^−^^3^
183 parameters	Extinction correction: *SHELXL2013* (Sheldrick, 2008)
0 restraints	Extinction coefficient: 0.0357 (16)
Primary atom site location: structure-invariant direct methods	Absolute structure: Flack *x* determined using 914 quotients [(*I*^+^)-(*I*^-^)]/[(*I*^+^)+(*I*^-^)] (Parsons *et al.*, 2013)
Secondary atom site location: difference Fourier map	Absolute structure parameter: 0.06 (4)
Hydrogen site location: inferred from neighbouring sites	

### Special details

**Table:**

Refinement. Refinement was performed using all reflections. The weighted *R*-factor (*wR*) and goodness of fit (*S*) are based on *F*^2^. *R*-factor (gt) are based on *F*. The threshold expression of *F*^2^ > 2.0 σ(*F*^2^) is used only for calculating *R*-factor (gt).

### Fractional atomic coordinates and isotropic or equivalent isotropic displacement parameters (Å^2^)

**Table:**

	*x*	*y*	*z*	*U*_iso_*/*U*_eq_	
O1	0.60120 (16)	0.50832 (14)	0.65955 (9)	0.0380 (4)	
O2	0.22423 (15)	0.29086 (15)	0.81979 (8)	0.0354 (3)	
O3	0.28367 (17)	0.34228 (17)	0.49573 (8)	0.0435 (4)	
O4	0.32128 (15)	0.66161 (14)	0.78685 (8)	0.0353 (3)	
O5	0.08071 (16)	0.6519 (2)	0.84322 (9)	0.0498 (4)	
N1	0.39627 (16)	0.38300 (15)	0.71946 (9)	0.0246 (3)	
N2	0.48080 (19)	0.24502 (17)	0.57047 (10)	0.0342 (4)	
C1	0.7414 (2)	0.4824 (2)	0.84759 (13)	0.0358 (4)	
C2	0.7647 (2)	0.4539 (2)	0.93501 (13)	0.0416 (5)	
C3	0.6535 (2)	0.3840 (2)	0.98308 (13)	0.0395 (5)	
C4	0.5124 (2)	0.3393 (2)	0.94584 (11)	0.0326 (4)	
C5	0.4892 (2)	0.36838 (17)	0.85916 (11)	0.0255 (4)	
C6	0.6010 (2)	0.43761 (18)	0.81107 (11)	0.0274 (4)	
C7	0.5420 (2)	0.45217 (18)	0.72098 (11)	0.0266 (4)	
C8	0.3518 (2)	0.33864 (17)	0.80233 (11)	0.0250 (4)	
C9	0.2867 (2)	0.38767 (19)	0.64668 (11)	0.0269 (4)	
C10	0.3548 (2)	0.32287 (19)	0.56467 (11)	0.0281 (4)	
C11	0.2218 (2)	0.5328 (2)	0.62906 (12)	0.0337 (4)	
C12	0.1075 (2)	0.5854 (2)	0.69751 (13)	0.0396 (5)	
C13	0.1827 (2)	0.63585 (19)	0.77980 (12)	0.0311 (4)	
H1	0.81748	0.52994	0.8148	0.0429*	
H2	0.85847	0.48296	0.96185	0.0499*	
H3	0.67296	0.36605	1.04192	0.0475*	
H4	0.43628	0.2915	0.97838	0.0392*	
H5	0.19512	0.32999	0.66336	0.0323*	
H6B	0.51794	0.20427	0.52459	0.0513*	
H7A	0.52731	0.23407	0.62019	0.0513*	
H8A	0.31049	0.59737	0.62494	0.0404*	
H9B	0.16801	0.53233	0.5729	0.0404*	
H10A	0.04576	0.66122	0.67253	0.0475*	
H11B	0.03387	0.51082	0.71193	0.0475*	
H12	0.1288	0.66053	0.8897	0.0747*	

### Atomic displacement parameters (Å^2^)

**Table:**

	*U*^11^	*U*^22^	*U*^33^	*U*^12^	*U*^13^	*U*^23^
O1	0.0368 (7)	0.0480 (8)	0.0294 (7)	−0.0115 (7)	0.0044 (6)	0.0064 (6)
O2	0.0285 (7)	0.0478 (8)	0.0299 (7)	−0.0078 (6)	0.0024 (6)	0.0079 (6)
O3	0.0411 (8)	0.0673 (10)	0.0221 (7)	0.0098 (8)	−0.0079 (6)	−0.0063 (6)
O4	0.0307 (7)	0.0439 (7)	0.0313 (7)	−0.0037 (6)	−0.0003 (6)	−0.0053 (6)
O5	0.0331 (7)	0.0856 (12)	0.0306 (8)	−0.0014 (8)	0.0020 (6)	−0.0085 (8)
N1	0.0237 (7)	0.0314 (7)	0.0188 (7)	−0.0022 (6)	−0.0007 (6)	0.0021 (6)
N2	0.0352 (9)	0.0442 (9)	0.0233 (8)	0.0063 (7)	−0.0016 (7)	−0.0053 (7)
C1	0.0269 (9)	0.0432 (10)	0.0373 (11)	−0.0013 (9)	−0.0037 (8)	−0.0049 (9)
C2	0.0322 (10)	0.0538 (12)	0.0388 (11)	0.0062 (10)	−0.0146 (9)	−0.0102 (10)
C3	0.0436 (12)	0.0508 (11)	0.0243 (10)	0.0134 (10)	−0.0094 (9)	−0.0036 (9)
C4	0.0362 (10)	0.0392 (10)	0.0225 (9)	0.0074 (9)	0.0008 (8)	0.0014 (8)
C5	0.0262 (9)	0.0286 (9)	0.0219 (9)	0.0046 (7)	0.0000 (7)	−0.0023 (7)
C6	0.0257 (8)	0.0315 (8)	0.0251 (9)	0.0030 (8)	−0.0013 (7)	−0.0024 (8)
C7	0.0239 (8)	0.0315 (8)	0.0244 (9)	−0.0024 (7)	0.0012 (7)	0.0001 (8)
C8	0.0272 (9)	0.0268 (8)	0.0210 (8)	0.0026 (7)	0.0004 (7)	0.0023 (7)
C9	0.0242 (8)	0.0364 (9)	0.0203 (9)	−0.0034 (7)	−0.0031 (7)	0.0016 (8)
C10	0.0259 (9)	0.0366 (9)	0.0217 (9)	−0.0053 (8)	−0.0027 (7)	0.0003 (7)
C11	0.0376 (10)	0.0409 (10)	0.0226 (9)	0.0074 (9)	−0.0064 (8)	0.0017 (8)
C12	0.0318 (9)	0.0517 (12)	0.0351 (11)	0.0115 (9)	−0.0089 (9)	−0.0073 (9)
C13	0.0321 (10)	0.0321 (9)	0.0291 (10)	0.0051 (8)	−0.0018 (8)	0.0016 (8)

### Geometric parameters (Å, º)

**Table:**

O1—C7	1.203 (2)	C9—C10	1.527 (2)
O2—C8	1.207 (2)	C9—C11	1.532 (3)
O3—C10	1.238 (2)	C11—C12	1.522 (3)
O4—C13	1.206 (2)	C12—C13	1.503 (3)
O5—C13	1.316 (2)	O5—H12	0.830
N1—C7	1.405 (2)	N2—H6B	0.870
N1—C8	1.402 (2)	N2—H7A	0.870
N1—C9	1.460 (2)	C1—H1	0.940
N2—C10	1.310 (2)	C2—H2	0.940
C1—C2	1.393 (3)	C3—H3	0.940
C1—C6	1.387 (3)	C4—H4	0.940
C2—C3	1.378 (3)	C9—H5	0.990
C3—C4	1.396 (3)	C11—H8A	0.980
C4—C5	1.382 (2)	C11—H9B	0.980
C5—C6	1.378 (2)	C12—H10A	0.980
C5—C8	1.487 (2)	C12—H11B	0.980
C6—C7	1.486 (2)		
			
C7—N1—C8	111.52 (14)	O4—C13—C12	123.81 (17)
C7—N1—C9	123.92 (14)	O5—C13—C12	112.90 (16)
C8—N1—C9	122.81 (14)	C13—O5—H12	109.469
C2—C1—C6	117.00 (17)	C10—N2—H6B	120.005
C1—C2—C3	121.55 (19)	C10—N2—H7A	120.001
C2—C3—C4	121.08 (18)	H6B—N2—H7A	119.994
C3—C4—C5	117.28 (17)	C2—C1—H1	121.519
C4—C5—C6	121.54 (16)	C6—C1—H1	121.506
C4—C5—C8	130.11 (16)	C1—C2—H2	119.202
C6—C5—C8	108.34 (15)	C3—C2—H2	119.210
C1—C6—C5	121.54 (16)	C2—C3—H3	119.482
C1—C6—C7	129.83 (16)	C4—C3—H3	119.476
C5—C6—C7	108.63 (15)	C3—C4—H4	121.355
O1—C7—N1	124.67 (16)	C5—C4—H4	121.352
O1—C7—C6	129.86 (16)	N1—C9—H5	106.340
N1—C7—C6	105.47 (14)	C10—C9—H5	106.336
O2—C8—N1	124.22 (16)	C11—C9—H5	106.343
O2—C8—C5	130.12 (16)	C9—C11—H8A	108.681
N1—C8—C5	105.63 (14)	C9—C11—H9B	108.685
N1—C9—C10	112.66 (14)	C12—C11—H8A	108.682
N1—C9—C11	113.19 (15)	C12—C11—H9B	108.682
C10—C9—C11	111.39 (14)	H8A—C11—H9B	107.616
O3—C10—N2	122.91 (17)	C11—C12—H10A	108.474
O3—C10—C9	117.83 (16)	C11—C12—H11B	108.468
N2—C10—C9	119.20 (15)	C13—C12—H10A	108.470
C9—C11—C12	114.32 (16)	C13—C12—H11B	108.470
C11—C12—C13	115.22 (16)	H10A—C12—H11B	107.495
O4—C13—O5	123.27 (17)		
			
C7—N1—C8—O2	171.27 (15)	C4—C5—C6—C7	179.74 (15)
C7—N1—C8—C5	−6.74 (17)	C4—C5—C8—O2	5.8 (3)
C8—N1—C7—O1	−174.78 (15)	C4—C5—C8—N1	−176.37 (17)
C8—N1—C7—C6	5.92 (17)	C6—C5—C8—O2	−172.97 (16)
C7—N1—C9—C10	63.76 (19)	C6—C5—C8—N1	4.88 (17)
C7—N1—C9—C11	−63.74 (19)	C8—C5—C6—C1	178.32 (13)
C9—N1—C7—O1	−9.5 (3)	C8—C5—C6—C7	−1.38 (18)
C9—N1—C7—C6	171.19 (13)	C1—C6—C7—O1	−1.5 (3)
C8—N1—C9—C10	−132.59 (14)	C1—C6—C7—N1	177.71 (17)
C8—N1—C9—C11	99.91 (17)	C5—C6—C7—O1	178.13 (16)
C9—N1—C8—O2	5.8 (2)	C5—C6—C7—N1	−2.62 (18)
C9—N1—C8—C5	−172.20 (13)	N1—C9—C10—O3	−167.77 (14)
C2—C1—C6—C5	0.2 (3)	N1—C9—C10—N2	15.1 (2)
C2—C1—C6—C7	179.85 (16)	N1—C9—C11—C12	−70.37 (18)
C6—C1—C2—C3	0.2 (3)	C10—C9—C11—C12	161.47 (13)
C1—C2—C3—C4	−0.2 (3)	C11—C9—C10—O3	−39.3 (2)
C2—C3—C4—C5	−0.1 (3)	C11—C9—C10—N2	143.54 (15)
C3—C4—C5—C6	0.5 (3)	C9—C11—C12—C13	77.4 (2)
C3—C4—C5—C8	−178.13 (16)	C11—C12—C13—O4	15.2 (3)
C4—C5—C6—C1	−0.6 (3)	C11—C12—C13—O5	−166.20 (15)

### Hydrogen-bond geometry (Å, º)

**Table:**

*D*—H···*A*	*D*—H	H···*A*	*D*···*A*	*D*—H···*A*
O5—H12···O3^i^	0.83	1.80	2.6230 (19)	172
N2—H6*B*···O3^ii^	0.87	2.32	2.891 (2)	123
N2—H7*A*···O4^iii^	0.87	2.05	2.886 (2)	161

Symmetry codes: (i) −*x*+1/2, −*y*+1, *z*+1/2; (ii) *x*+1/2, −*y*+1/2, −*z*+1; (iii) −*x*+1, *y*−1/2, −*z*+3/2.
